# MergeAlign: improving multiple sequence alignment performance by dynamic reconstruction of consensus multiple sequence alignments

**DOI:** 10.1186/1471-2105-13-117

**Published:** 2012-05-30

**Authors:** Peter W Collingridge, Steven Kelly

**Affiliations:** 1 Marine Biological Association of the United Kingdom, The Laboratory, Citadel Hill, Plymouth, PL1 2PB, Devon, UK; 2 Department of Plant Sciences, University of Oxford, South Parks Road, OX1 3RB, Oxford, UK; 3 Centre for Mathematical Biology, Mathematical Institute, University of Oxford, 24-29 St Giles’, OX1 3LB, Oxford, UK; 4 Oxford Centre for Interactive Systems Biology, Department of Biochemistry, University of Oxford, South Parks Road, OX1 3QU, Oxford, UK; 5 Sir William Dunn School of Pathology, University of Oxford, South Parks Road, OX1 3RE, Oxford, UK

## Abstract

**Background:**

The generation of multiple sequence alignments (MSAs) is a crucial step for many bioinformatic analyses. Thus improving MSA accuracy and identifying potential errors in MSAs is important for a wide range of post-genomic research. We present a novel method called MergeAlign which constructs consensus MSAs from multiple independent MSAs and assigns an alignment precision score to each column.

**Results:**

Using conventional benchmark tests we demonstrate that on average MergeAlign MSAs are more accurate than MSAs generated using any single matrix of sequence substitution. We show that MergeAlign column scores are related to alignment precision and hence provide an *ab initio* method of estimating alignment precision in the absence of curated reference MSAs. Using two novel and independent alignment performance tests that utilise a large set of orthologous gene families we demonstrate that increasing MSA performance leads to an increase in the performance of downstream phylogenetic analyses.

**Conclusion:**

Using multiple tests of alignment performance we demonstrate that this novel method has broad general application in biological research.

## Background

The construction of multiple sequence alignments (MSAs) from individual sequences is fundamental to nearly all aspects of post-genomic biological research. In addition to the role these alignments play in progressing our understanding of the evolution and diversity of life, they also provide a platform from which algorithms that predict protein structure and function can be based. Given their pivotal role, the development of improved MSA algorithms and matrices of sequence evolution has been an active area of research for more than 20 years. The result of this research has been the production of many different MSA methods whose performances on diverse data types can vary considerably (for example of comparative analyses of method performance see [1,2]).

The rationale for the continued improvement of alignment methods is that reduction in error in multiple sequence alignment will thus lead to reduction in error in all subsequent downstream bioinformatic analysis. For example, in phylogenetic analysis it has been demonstrated that alignment error leads to tree inference error regardless of the inference method used downstream of alignment construction [3]. In addition to reducing error by improving alignment accuracy, independent methods have been developed to identify and discard alignment data which may contain errors. As information gaps are a major source of alignment error a common approach adopted in phylogenetic analysis is to discard information when it exceeds a threshold value of gap-characters. Popular methods such as GBLOCKS [4] have been developed to automate this error reduction process and thereby reduce the amount of possibly erroneous data in MSAs. These strategies are hard-lined with regard to gap characters and hence even correctly aligned insertion and deletion events can be discarded. Due to the high occurrence of insertions and deletions in real biological sequence data, (for example the Pfam [5] seed alignments of conserved domains contain approximately 38.6% gap-characters) the danger of gap parsing is that one can reduce the usable phylogenetic information to a level which can be insufficient to facilitate a robust resolution of phylogenetic relationships. Moreover, some phylogenetic inference methods ignore or misinterpret gaps as additional character states which can lead to errors in phylogenetic inference. As an alternative strategy to discarding gap-rich data methods such as PRANK [6,7] have been developed which incorporate the phylogenetic implications of gaps and treat insertions and deletions as separate evolutionary events.

It has been demonstrated that for some MSAs, different methods correctly align different regions whilst no current method correctly aligns the entire sequence [2]. This work showed that it is possible to improve the accuracy of MSAs by creating consensus MSAs based on multiple independent MSAs of the same sequence [8,9]. M-COFFEE is such a meta-method which uses MSAs generated by individual MSA methods to generate consensus alignments that are more accurate, on average, than any of the individual alignment methods used [9].

Here, we provide a novel algorithm called MergeAlign that uses a dynamic programming approach to efficiently construct consensus MSAs from any number of independent alignments of the same sets of sequences. To generate input MSAs for this method we use a single alignment methodology and multiple different matrices of amino acid substitution. We use standard alignment benchmark tests to show that the consensus alignments produced in this way are more accurate than those produced using any individual matrix of amino acid substitution. We further show that our method is suitable for combining alignments generated using different methodologies. Creating consensus alignments from large numbers of constituent alignments allows us to assign a score to each column in the final MSA. We show that the MergeAlign column score is related to alignment error rate and hence provide a novel method for data selection based on expected alignment error rate. In addition to improving performance on MSA benchmarks we demonstrate that MergeAlign alignments improve performance of downstream phylogenetic analysis. The MergeAlign algorithm is provided as Additional file 1 and is available at http://www.mergealign.com.

## Results and discussion

### A novel method for constructing the consensus of multiple sequence alignments

**Figure 1:**
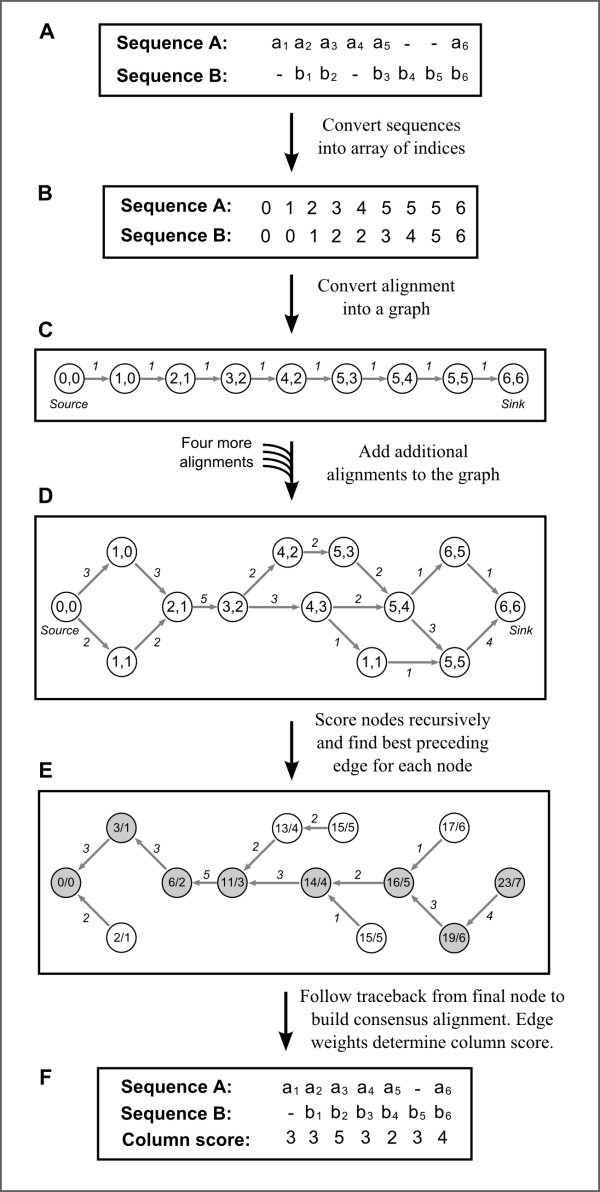
**Cartoon of the MergeAlign algorithm.** In this example, MergeAlign generates a consensus alignment of two sequences **A** and **B**, based on five independent MSAs. **(A)** An example of one of the five constituent MSAs. **(B)** The numerical representation of the MSA in (A). **(C)** A graph of the alignment in (A), using the columns of (B) to identify nodes. All edge weights are equal to 1 because only one alignment has been considered. **(D)** The graph after 4 more MSAs have been added. **(E)** Each node is given two values: *path score*/*path length*. Both are set to 0 for the sink nodes and other nodes are scored recursively. **(F)** By following the traceback path, the optimum consensus alignment is reconstructed. Each column of the MSA is given a score equal to the weight of the corresponding edge. A full description of the algorithm is given in the methods.

We developed a novel method for constructing consensus multiple sequence alignments (MSAs) from any number of input multiple sequence alignments. In brief, we create a weighted directed acyclic graph that represents each of the constituent MSAs (Figure 1). In this graph, each node represents a column found in at least one of the constituent MSAs and each edge represents the transition between two columns. The weight of each edge is equal to the number of MSAs that contain that transition. A dynamic programming approach is then utilised to find the path through the graph that maximises the mean weight of the edges traversed (Figure 1). This path is converted into a consensus MSA and the edge weights used to score each column.

### Multiple substitution matrices achieve near equivalent performance on alignment benchmarks

**Figure 2:**
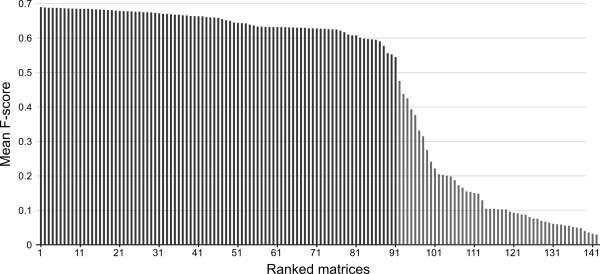
**Mean F-score of MSAs generated using 142 amino acid substitution matrices.** MSAs were generated from 1000 randomly selected benchmark MSAs and inferred using MAFFT FFT-NS-2. Substitution matrices were ranked based on their mean F-score for this test set of benchmark MSAs. Based on these results, the first 91 top-ranked substitution matrices (all those scoring above a threshold of 0.5, shown in dark grey), were used for subsequent analyses.

To generate a set of input alignments for use for consensus MSA construction we chose to adopt a novel approach which involves creating multiple MSA using different matrices of amino acid substitution. We first assayed the MSA performance of a set of 142 previously characterised amino acid substitution matrices to identify how each substitution matrix performed on benchmark MSAs. The MAFFT [10] MSA method was used with the FFT-NS-2 strategy to align each of a randomly selected set of 1000 benchmark sequences ( Additional file 2), using each amino acid substitution matrix. The F-score for each alignment was determined by comparison of the inferred alignment to the benchmark MSA, which is assumed for this purpose to represent the true alignment (see methods). The amino acid substitution matrices were then ranked according to the mean F-score obtained over all MSAs (Figure 2, Additional file 3).

This analysis identified JOHM930101 as the highest scoring substitution matrix with a mean F-score of 0.6885 over the 1000 randomly selected benchmark MSAs. Interestingly this matrix did not obtain significantly higher scores than the next 10 best substitution matrices assayed by paired *t*-test (*p* <0.01). Moreover, the F-score of the first 91 matrices decreased approximately linearly with each subsequently ranked matrix (Figure 2). Beyond the substitution matrix ranked 91^st^, the mean F-score of subsequent matrices reduced rapidly. We therefore selected these top-scoring 91 amino acid substitution matrices for generating input alignments for the MergeAlign method.

### MergeAlign alignments are more accurate that alignments based on any individual amino acid substitution matrix

In order to test how MergeAlign consensus MSAs perform compared to MSAs generated with a single substitution matrix, we selected a second set of 1000 randomly selected benchmark MSAs ( Additional files 4 &5). We aligned each member of this set using both MAFFT FFT-NS-2 and the more accurate MAFFT L-INS-i method [10]. Individual MSAs were generated for both methods using each of the 91 top-scoring substitution matrices from the previous test. The mean F-score observed using each substitution matrix is highly correlated between FFT-NS-2 and L-INS-i alignment methods and the rank order of amino acid substitution matrices is near identical (Spearman r = 0.959, *p* < 1 × 10^−10^Additional file 5). Having selected 91 MSAs for each benchmark MSA, we used MergeAlign to generate a consensus of these individual MSAs. Consensus MergeAlign MSAs were generated from all 91 MSAs or subsets of the 91 MSAs. Subsets consisted of those MSAs generated by the top 10, or multiples of 10 up to 80, substitution matrices according to rank order of the mean F-score shown in Figure 2. We scored each MSA, including the MergeAlign consensus MSAs, using the mean F-score of the 1000 independent MSA inferences.

**Figure 3:**
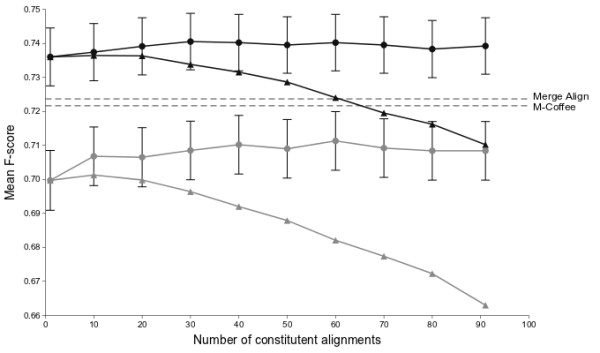
**Effect of generating MergeAlign consensus MSAs with increasing numbers of constituent alignments.** Individual MSAs were generated for a set of 1000 randomly selected benchmark MSAs using either MAFFT FFT-NS-2 (grey lines) or MAFFT L-INS-i (black lines). MSAs were added to the consensus MSA in the order in which the matrices that generated them are ranked ( Additional file 2). As a result, the mean F-score of constituent MSAs (marked with triangles) drops as more alignments are included. However, the mean F-score of MSAs generated by MergeAlign (marked with circles) is relatively stable. Furthermore, MergeAlign outperforms M-Coffee (dashed line) on the same set of benchmark MSAs.

The MSAs generated by MergeAlign scored consistently high, whether using MSAs generated from all 91 amino acid substitution matrices or a subset of them (Figure 3, black circles). As expected the average score of the constituent MSAs generally decreased as more alignments were included, however the score of the consensus alignments made by MergeAlign did not follow the same trajectory. For example, when using FFT-NS-2 (Figure 3, grey circles) the mean F-score for MergeAlign ranged from 0.7065 for a consensus of 20 MSAs to 0.7113 for a consensus of 60 MSAs while the mean F-score of those 60 MSAs was only 0.6821. All of the MergeAlign consensus MSAs also scored higher than the MSAs generated by the previously identified top scoring substitution matrix (JOHM930101; mean F-score = 0.7045). When using the L-INS-I method to generate input MSA for MergeAlign, the MergeAlign method also outperformed all individual substitution matrices, though the effect was less pronounced (Figure 3, solid black lines). The maximum F-score for MergeAlign was 0.7405, which was obtained by generating a consensus MSA of the top 30 ranked substitution matrices identified by the training set.

To provide a direct comparison to an existing method which produces a consensus from MSAs in a different way, we aligned the 1000 benchmark MSAs above with M-Coffee [9]. Here, M-Coffee creates a consensus alignment using 8 independent MSA methods [9]. To compare the methods directly the same 8 constituent MSAs were also combined using MergeAlign. Starting from the same raw input alignments the mean time taken to construct a consensus alignment was 8.60 s (standard deviation 16.362 s) and 0.10 s (standard deviation 0.164 s) for M-Coffee and MergeAlign respectively. The mean score for M-Coffee was 0.7217 (Figure 3) whereas the mean score for MergeAlign was 0.7237 (Figure 3). Thus on small numbers of input alignments MergeAlign is significantly faster than M-Coffee (*p* < 1 × 10^−100^) however the accuracy of the two methods is not significantly different (*p* = 0.253).

**Figure 4:**
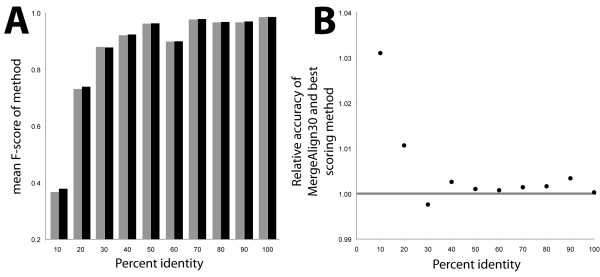
**Performance of MergeAlign on benchmark alignments by %id of sequences in reference alignments. A)** Grey columns represent top scoring matrix of amino acid substitution (JOHM930101), black columns represent score of MergeAlign constructed from the top 30 ranked substitution matrices. **B)** Relative percentage difference between columns in A.

Analysis of the accuracy of all tested MSA methods as a function of the percentage identity of the reference MSA revealed that MergeAlign outperforms all tested methods across nearly all levels of sequence identity (Figure 4A&B). Interestingly, the most pronounced improvement in accuracy occurs for sequences with low percentage identity (Figure 4B). For the lowest category (i.e. 0–10% sequence identity) MergeAlign achieves ~3% improvement in accuracy. The difference between the accuracy of MergeAlign and other methods is less pronounced at higher levels of sequence identity (Figure 4B).

### MergeAlign column score is related to alignment accuracy

**Figure 5:**
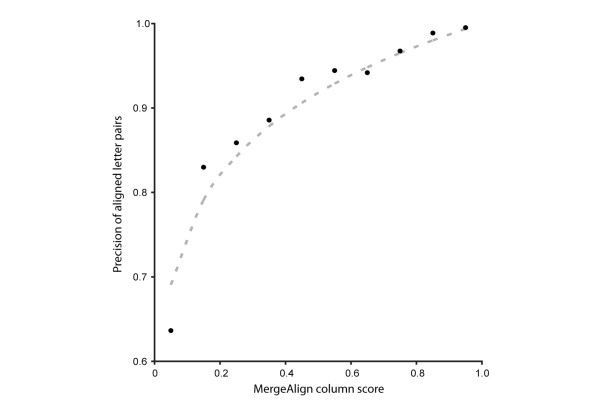
MergeAlign column score is related to alignment precision.

When MergeAlign generates a consensus MSA from multiple input MSAs it assigns each column a score equal to the proportion of constituent MSAs in which that column is present. To determine the relationship between MergeAlign column score and MSA error rate, the alignment precision and MergeAlign column score for all aligned columns were compared. The individual columns from all MSAs inferred using MergeAlign were binned into categories based on their MergeAlign column score with a score bin width of 0.1. For each category, the mean precision of the aligned letter-pairs for the constituent columns was evaluated. The relationship between the MergeAlign column score and the precision of the aligned letter pairs contained within that column was fit to a power function of the form f(x) = x^m^. This was subject to least squares fitting and a value of 0.124 was selected as the optimal value for m (Figure 5). This analysis revealed that MergeAlign column score is related to alignment precision and hence, one can estimate the error rate of an individual column in a MergeAlign MSA as a function of its MergeAlign column score. For example, columns obtaining a MergeAlign column score of ≥ 0.92 will have an expected false positive alignment error rate of ~1%. Similarly columns obtaining a MergeAlign column score of 0.66 will have an expected false positive error rate of ~5%.

### Increasing alignment F-score on benchmark tests improves phylogenetic tree performance

**Figure 6:**
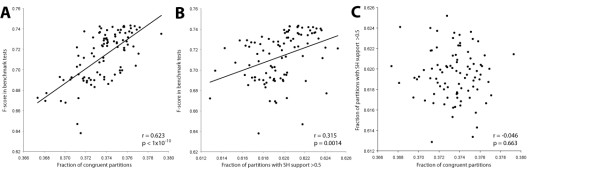
**Comparison of multiple sequence alignment tests. A)** Comparison of topological agreement test with F-score from alignment benchmark test. **B)** Comparison of topological support test with F-score from alignment benchmark test. **C)** Comparison of topological agreement and topological support tests.

As the use of MSA benchmarks for evaluating MSA performance has recently been called into question [1] we developed an alternative test to validate that MergeAlign MSAs were better than those generated by single matrices of amino acid substitution. This test involved measuring the effect of the MSA method on two aspects of phylogenetic tree inference. 1) The topological agreement between single gene phylogenetic trees obtained from the same set of organisms. 2) The fraction of partitions in those trees which received a SH-like support value of >0.5. A large set of single gene orthologous protein families from the same set of organisms were downloaded from a previous analysis [11]. Each of these sequence families containing four or more sequences (N = 1688) was aligned using each of the 91 substitution matrices selected above. MergeAlign alignments were constructed from subsets of the 91 alignments using the rank order derived from the benchmark test as described before. Each alignment was then used to infer a phylogenetic tree and the degree of topological agreement between trees was calculated. Comparison of the topological agreement with F-score from the benchmark tests revealed that there is a significant positive correlation between these two scores (Figure 6A, r = 0.623, *p* < 1 × 10^−10^). There is also positive (but less significant) correlation between the fraction of partitions which receive a SH-like support of >0.5 and the F-score in the benchmark tests (Figure 6B, r = 0.315, *p* = 0.0014). There was no significant correlation between the fraction of partitions which receive a SH-like support of >0.5 and the level of topological agreement between trees (Figure 6C, r = −0.046, *p* = 0.663) hence these two tree-based tests can be considered to be independent. This analysis demonstrates that substitution matrices which performed better in MSA benchmark tests produce trees which are both more resolved and more consistent with each other.

### Merge Align improves performance on structural measure of multiple sequence alignment accuracy

To determine whether MergeAlign also performs well on structural measures of multiple sequence alignment accuracy the benchmark multiple sequence alignments were subject to benchmark free accuracy evaluation using the iRMSD method [12]. This analysis revealed that Merge Align also scored very highly using this method of evaluation (Table 1, Additional file 5). To put this result in context and evaluate and compare the performance of MergeAlign across all tests the score obtained in each test was ranked and the average rank across all tests was calculated (Table 1). This revealed that on average across all tests presented MergeAlign using all 91 input alignments was the highest ranked method.

**Table 1:** Performance of top scoring methods across all alignment performance tests

**Method**	**Rank in Benchmark Test**	**Rank in Tree Resolution Test**	**Rank in Topological Congruence Test**	**Rank in Structural Accuracy Test**	**Mean rank**
MergeAlign91	8	20	2	9	9.8
MergeAlign80	9	8	9	14	10.0
MergeAlign70	5	14	17	5	10.3
MergeAlign30	1	23	10	11	11.3
MergeAlign60	3	31	6	8	12.0
CROG050101	13	5	29	6	13.3
CombAlign40	2	32	20	4	14.5
DOSZ010104	11	44	4	1	15.0
MergeAlign50	7	26	21	10	16.0
OVEJ920101	20	25	8	12	16.3
JOHM930101	21	12	15	18	16.5
MergeAlign20	6	11	37	13	16.8
BLOSUM80	22	22	1	24	17.3
BLOSUM62	18	10	27	22	19.3
MergeAlign10	10	6	57	7	20.0
PRLA000102	12	33	24	19	22.0
CSEM940101	24	15	47	3	22.3
DOSZ010103	4	27	60	2	23.3
PRLA000101	17	9	59	15	25.0
HENS920103	26	34	12	32	26.0

## Conclusion

We present a novel method for improving multiple sequence alignment (MSA) performance by constructing consensus MSAs from multiple individual MSAs. In all tests performed on benchmark MSAs the precision, recall (Q-score) and iRMSD score of the MergeAlign method is better than that observed for any individual matrix of amino acid substitution. This is the first demonstration that combining of MSAs using different models of amino acid substitution can improve MSA accuracy. We also demonstrated in two independent tests that did not use benchmark MSAs that MergeAlign alignments increase the resolution of phylogenetic trees and increase the topological agreement between phylogenetic trees inferred from individual gene families.

This method for improving MSA has broad general applicability in biological research. The method is also extensible: as more matrices of sequence substitution become available they can be incorporated into the MergeAlign procedure. Similarly, MergeAlign will benefit from improvements in underlying MSA technology; as these improve MergeAlign will improve. Independent of its use for inferring consensus MSAs, MergeAlign is also suitable for identifying the consensus columns between MSAs generated under different alignment methods. MergeAlign also provides an estimate of the precision of a MSA on a column-by-column basis. We demonstrated that the MergeAlign column score is related to MSA precision and propose this as an additional and independent method for data selection for downstream bioinformatic applications.

## Methods

### Constructing MergeAlign consensus multiple sequence alignments

In order to construct consensus MSAs we create a weighted directed acyclic graph that represents all the constituent MSAs (Figure 1). In this graph, each node represents a column present in at least one constituent MSA; each edge represents the transition between two columns in at least one constituent MSA; each edge weight represents the number of MSAs containing that transition. We then use a dynamic programming approach to find the path through the graph, from source vertex to sink vertex, that maximises the mean weight of the edges traversed (Figure 1). This path is converted into a consensus MSA and the edge weights used to score each column.

In order to identify columns common between MSAs, we first convert each sequence within each alignment into an array of indices (Figure 1). This is done by replacing the letter representing each amino acid with a number representing that amino acid’s position in the sequence and by replacing each gap character by the number of the preceding amino acid. All sequences are assumed to start with a 0. Each column in an MSA can then be converted into an *N*-tuple, where *N* is the number of sequences in that MSA. Once a graph has been constructed, each node is given a path score and a path length. The path score and path length of the source node are set at zero and all other nodes are scored recursively as follows. With the exception of the source node, each node *n*, has at least one incoming edge, *e*_*i*_ = (*x*_*i*_, *n*), where *x*_*i*_ is the source of that edge. Each incoming edge, *e*_*i*_, is given a score equal to:

(1) $$\frac{s\left(x_{i}\right)+w\left(e_{i}\right)}{l\left(x_{i}\right)+1}$$

where *s*(*x*_*i*_) is the path score of the node *x*_*i*_, *l*(*x*_*i*_) is the path length of node *x*_*i*_, and *w*(*e*_*i*_) is the weight of edge *e*_*i*_. The edge with the highest score, *e*_*s*_ = (*x*_*s*_, *n*), is then selected. The path score of node *n* is set to *s*(*x*_*s*_) + *w*(*e*_*s*_), and the path length of node *y* is set to (*x*_*s*_) + 1 (See Figure 1 E; nodes are labelled with *path score*/*path length*). A traceback edge *t* = (*n*, *x*_*s*_) is created with the same weight as *e*_*s*_ for the traceback process. The path score of any node is thus the maximum sum of edge weights for a path from the source node to that node; the path length is the length of this optimal path. The optimum path from source to sink is found by starting at the sink node and following the traceback edges. This path is used to reconstruct the consensus MSA while the weights of the traceback edges are used to score each column. The column score can be normalised by dividing it by the total number of input MSAs. A python implementation of the above algorithm is presented as Additional file 1 and is available online at http://www.mergealign.com.

Since MergeAlign only considers columns present in constituent MSAs it is very efficient and scalable. Generating a consensus MSA for 834 unique benchmark MSAs (on average consisting of 37 sequences each 256 amino acids long) from 91 constituent MSAs took an average of 1.1 s per consensus MSA on one processor of a standard laptop. A graph built by MergeAlign has a maximum of *kN* vertices, where *k* is the number of constituent MSAs, and *N* is the maximum number of columns in any constituent MSA. The maximum number of edges is entering or leaving a vertex is *k*. The speed at which MergeAlign can build and traverse the graph is therefore given by O(*Nk*^*2*^), thus linear with respect to MSA length and independent of the number of sequences in an MSA.

This method can be viewed as an extension of the classical dynamic programming approach used to construct pairwise sequence alignments [13,14]. These algorithms represent all possible alignments of two sequences as a 2D array and use dynamic programming to find the optimum path through the matrix. These methods require scoring every possible column in an MSA and hence run in O(*N*^*k*^) time, thus rendering them unusable for more than a few sequences [15]. MergeAlign is able to extend this approach to MSA by limiting its search to only columns present in one or more constituent alignment.

### Amino acid substitution matrix and benchmark MSAs

The source data used in this work comes from a number of publicly available databases. A set of 137 amino acid substitution matrices was downloaded from AAindex http://www.genome.jp/aaindex/[16]. This set comprised amino acid substitution matrices and statistical protein contact potentials derived from different source datasets using a variety of different methods described in [16]. The matrix VOGG950101 was removed from this set because it resulted in alignments which were identical to those generated when using matrix GONG920101. In addition to these matrices we also included commonly used matrices which were absent from this list including BLOSUM62 (as used by BLAST [17]), BLOSUM80, BLOSUM90, PAM30, PAM70 and VTML200. This resulted in a final set of 142 substitution matrices ( Additional file 3). Benchmark MSAs were obtained from http://www.drive5.com/bench. These comprised the complete set of benchmark MSAs from Balibase v3 [18], OXBENCH [19], PREFAB v4 [20] and SABRE [20,21]. In total this set contained 2724 benchmark MSAs.

### Substitution matrix selection and alignment inference

To select the optimal substitution matrices for use in MergeAlign a random subset of 1000 benchmark MSAs was sampled with replacement from the complete set of 2724. This set contained 804 different benchmark MSAs ( Additional file 2). This training set of sequences were un-aligned and MSAs were inferred for each member using each of the 142 amino acid substitution matrices above. MSAs were generated using MAFFT [10] with the FFT-NS-2 method, which has been shown to be as accurate as the clustalw method [2,22]. Different matrices of amino acid substitution were utilised by MAFFT by using the “--aamatrix” option and specifying one of the matrices listed in Additional file 2 described above.

### Assessment of MSA performance

Each MSA inferred in this work was compared to its corresponding benchmark reference MSA and the number of true positive (TP) false positive (FP) and false negative (FN) aligned letter pairs were recorded. TP is the number of correctly aligned letter-pairs. FP is the number of aligned letter-pairs present in the inferred MSA that are not found in the benchmark MSA. FN is the number aligned letter-pairs in the benchmark MSA that are not found in the inferred MSA. These scores were then used to evaluate the precision and recall of the inferred MSAs where

(2) $$Precision=\frac{TP}{TP+FP}$$

(3) $$Recall=\frac{TP}{TP+FN}$$

The F-score was evaluated as the harmonic mean of the above precision and recall scores. The recall score presented here is mathematically equivalent to the alignment Q-score which has been previously described [20]. Precision scores for each column in the inferred MSAs were also calculated. In this case each column is treated independently and only the aligned letter-pairs present in the inferred column were used to calculate the precision score as described above. The combination of precision and recall (Q-score) to generate the F-score hence represents an incremental improvement to existing scoring methods. A Perl algorithm which computes column-by-column precision scores as well as whole alignment precision, recall and F-scores is presented as Additional file 6. In all cases concerning MSA benchmarks the true alignment is not known and the benchmark alignments contain FP and FN errors. For the purposes of this test the benchmark alignment is assumed to be true. In support of these benchmark based scores an independent measure of accuracy derived from structural data is also provided using the iRMSD method [12]. An additional and independent test that does not involve benchmark alignments is provided below.

### Evaluation of MergeAlign MSA performance

To evaluate the performance of MergeAlign and compare it to MSAs inferred from single matrices of amino acid substitution a second, independent test set of 1000 benchmark MSAs were randomly selected with replacement from the original set of 2724. This test set contained 834 discrete benchmark MSAs ( Additional file 4). Each member of this test set was aligned using each substitution matrix using MAFFT and the FFT-NS-2 method. FFT-NS-2 is a fast progressive method which performs two iterations of tree-guided progressive multiple sequence alignment [10]. This method has been demonstrated to be about as accurate as the well established clustal methods [2,10], though significantly faster. To provide an additional test, MSAs were inferred for each benchmark MSA using the L-INS-i method with each substitution matrix. L-INS-I has been repeatedly demonstrated to be one of the most accurate MSA algorithms currently available [2,10]. In brief, this method has a different objective function to FFT-NS-2, which evaluates the consistency between pairwise alignments and global multiple sequence alignment [10]. In this method flanking sequences surrounding the alignable regions are ignored and iterative refinement is allowed to proceed for a maximum of 1000 cycles [10]. For each benchmark, MergeAlign consensus MSAs were constructed from alignments generated by either method. The performance of MergeAlign was assayed by calculating the F-score of the consensus MSAs as described above and comparing it to the F-score for each of the individual MSAs generated using a single substitution matrix. To provide a comparison with a similar, consensus, methodology the same set of 1000 benchmark MSAs was also aligned using M-Coffee [9].

To determine the relationship between MergeAlign column score and alignment accuracy the individual columns from all MSAs inferred using MergeAlign were grouped into categories based on their MergeAlign column score. For each category, the precision of the aligned letter-pairs for the constituent columns was evaluated using the method described above. To determine the relationship between the MergeAlign column score and MSA precision this data was then fit to a power function of the form *f(x) = x*^*m*^ using a least squares method.

### Benchmark-free MSA performance evaluation

To provide an additional and independent assay for MSA performance we developed a novel test based on increasing the performance of downstream phylogenetic analysis. To do this we selected a large dataset of orthologous gene families which comprises 3537 orthologous gene families differentially distributed across 48 species of Archaea [11], where each gene family has no more than one sequence representative per taxa. It is assumed that the majority of these gene families will share the same evolutionary history and hence a more accurate MSA should improve topological agreement between individual gene family phylogenetic trees. For each of the orthologous groups containing four or more sequences (n = 1688) we constructed a MSA using each of the 91 selected amino acid substitution matrices. MSAs were also constructed using MergeAlign and a subset of substitution matrices which were selected according to their ranked performance of the benchmark tests. The top ranked 10, 20, 30, 40, 50, 60, 70, 80 or all 91 substitution matrices were used to construct MergeAlign alignments. Each alignment was then subject to phylogenetic inference using the FastTree program [23] with CAT rates. The proportion of partitions in each tree which received greater than 0.5 support by SH-like test [23] were recorded. All possible pairwise comparisons between trees (without replication) were performed for each MSA method (n = 7.06x10^10^). For each pairwise comparison between two trees, both trees were pruned to contain the identical set of taxa using the dendropy python module [24]. Only those trees which had four or more taxa in common and only partitions which received SH support of >0.5 were used for the analysis. The two trimmed trees were then compared using the dendropy python module [24] and the fraction of bi-partitions which were in agreement was evaluated.

## Competing interests

The authors declare that they have no competing interests.

## Authors’ contributions

PWC and SK designed the experiments, carried out the analysis and drafted the manuscript. Both authors read and approved the final manuscript.

## Supplementary Material

Additional file 1 The MergeAlign python program. Click here for file

Additional file 2 List of training set of benchmark alignments. Click here for file

Additional file 3 Mean F-score of training set for all 142 matrices of amino acid substitution. Click here for file

Additional file 4 List of test set of benchmark alignments. Click here for file

Additional file 5 Results for all tests of multiple sequence alignment performance. Click here for file

Additional file 6 Perl script for evaluating precision, recall, and F-score of multiple sequence alignments. Click here for file
